# The potential influence of the ligament of Wieger on the crystalline lens shape

**DOI:** 10.1038/s41598-024-54674-w

**Published:** 2024-02-18

**Authors:** Hosna Ghaderi, Sorcha Ní Dhubhghaill, Marie-José Tassignon, Luc Van Os, Carina Koppen, Jos J. Rozema

**Affiliations:** 1 Visual Optics Lab Antwerp (VOLANTIS), Faculty of Medicine and Health Sciences, University of Antwerp, Wilrijk, Belgium; 2 Department of Ophthalmology, Brussels University Hospital, Brussels, Belgium; 3 Department of Ophthalmology, Antwerp University Hospital, Edegem, Belgium

**Keywords:** Biomedical engineering, Mechanical engineering, Optics and photonics

## Abstract

This research uses mathematical modelling to evaluate the influence of the ligament of Wieger on the crystalline lens shape at rest, and during accommodation. An axisymmetric model of the anterior segment, including the ligament of Wieger, was created using the finite element method. Different conditions including variations of stiffness and positions of the ligament, with and without the ligament, were tested to see how they affected lens curvature and optical power. Adding the ligament of Wieger to the simulation had a noticeable impact on the optical power of the lens, particularly on the posterior surface power and total power. Ligament stiffness and width significant influenced the accommodative range of the eye by − 0.95D and − 2.39D for ligaments with the same and *3*× the stiffness of the capsular bag, respectively. Ligament width and inner diameter had negligible effects on lens thickness but did have significant effects on posterior surface power and accommodation. In this simulation, we found that the ligament of Wieger can significantly affect the lens shape, both at rest and during accommodation, and may need to be considered in lens models.

## Introduction

Accommodation is the process by which the crystalline lens changes shape to adjust the focus of the eye and allow for clear vision at various distances. The most widely accepted explanation of the mechanism of accommodation was proposed by Helmholtz^1^, in which the ciliary muscle either stretches or relaxes the zonular fibers to change the lens shape and alter the focus. When the ciliary muscle contracts, it relaxes the tension on the zonular fibers and allows the lens to bulge. This induces an increase in both lens curvature and refractive power for near vision. Conversely, when the ciliary muscle relaxes the zonular fibers tighten, causing the lens to become flatter and lower its focusing power for improved far vision. This is highlighted by a computer simulation by Goldberg^2^ of how the synchronized movements of the ciliary body and lens during accommodation, which led to a new theory of reciprocal zonular action^2^. Subsequent research examining the influence of zonular fibers on accommodation revealed that the anterior and equatorial zonules play significant roles in modulating the lens's optical power^3^. Specifically, it was observed that the anterior zonular fibers exert a negative effect, while the equatorial fibers contribute positively to the accommodation process. But despite the recent leaps in understanding on the mechanics of accommodation^4–6^, there is still much to be learned about the complex interactions that affect the lens shape.

One of the lesser-known anatomical features of the lens is the ligament of Wieger, an annular structure attached to the posterior lens capsule first described by Germain Wieger^7^. The existence of this ligament and its association with the posterior lens capsule were later confirmed by Mortada^8^, Campanella^9^, and visualized using optical coherence tomography (OCT) by Tassignon^10^. Ljubimova et al.^11^ studied the influence of the vitreous on accommodation and included the ligament of Wieger into their analysis, indicating that the ligament dampens the changes in the posterior lens curvature. The presence of the ligament was therefore thought to affect the tension of the lens capsule and the overall accommodation^11,12^. But as Ljubimova et al. did not investigate the impact of the position or stiffness of the Wieger ligament on the lens shape in their models, this aspect remains to be investigated. To this end, current work aims to use the Finite Element Method (FEM) to simulate the interactions between the ligament of Wieger and the rest of the lens anatomy as a function of ligament size and stiffness, and to examine how this affects lens reshaping during accommodation.

## Method

### Geometry

An axisymmetric FEM model of the entire eye was developed in ANSYS Mechanical 2023R2 (Ansys Inc, Southpointe, PA, USA), which included the cornea, limbus, sclera, iris, zonular fibers, ciliary muscles, crystalline lens, lens capsule, and ligament of Wieger, which aligns with previously published models for a 29-year-old eye^5,6,13^. Similar to Ljubimova^11^, the ligament of Wieger was assumed to follow the shape of the posterior surface of the lens. The lenticular cortex and nucleus are considered separately, as they both contribute to the lens overall power and shape, and their inclusion can provide more detailed insight into the lens reshaping^4,14^.

**Figure 1:**
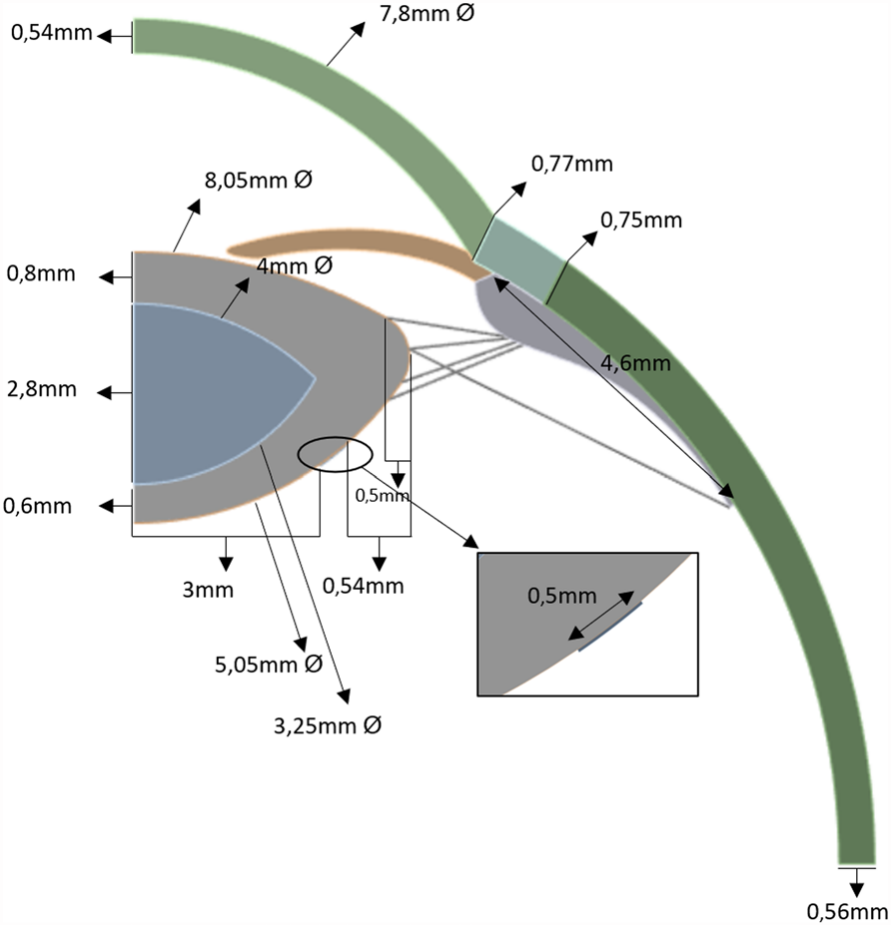
Schematic of the lens geometry; inset: Insertion point of the ligament of Wieger.

**Figure 2:**
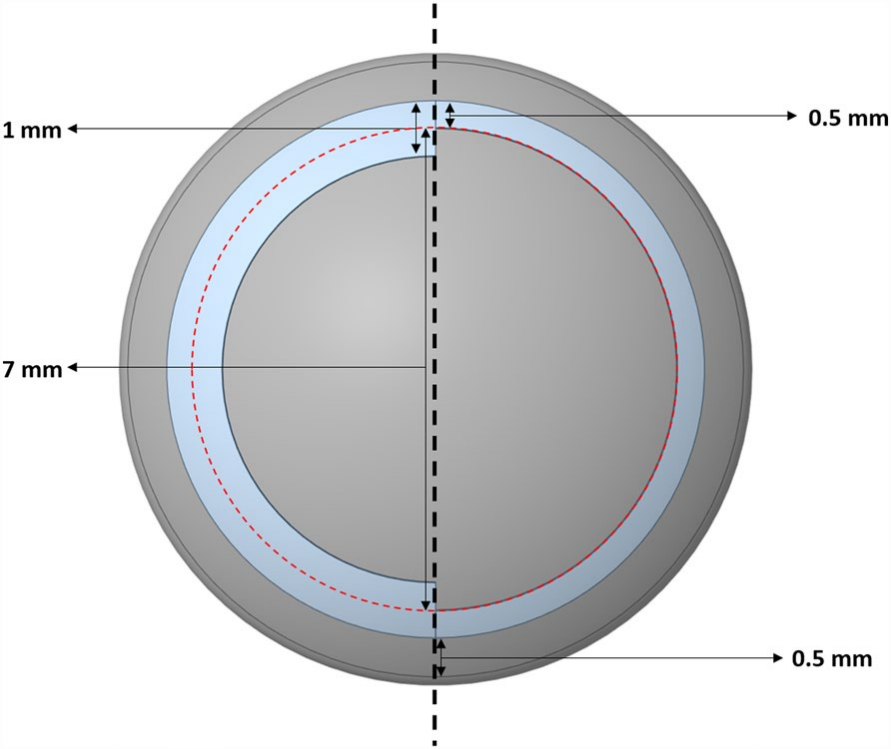
3D schematic of the posterior lens surface. Left: ligament of 0.5 mm or 1 mm width and a 6 mm inner diameter; right: ligament of 0.5 mm width and a 7 mm inner diameter.

The cornea, sclera, iris, ciliary muscles, and crystalline lens were modelled in the accommodated state, including the cortex and nucleus (Table 1), while the lens capsule was considered as a surface coating of 10 µm in thickness^6^. The zonular fibers were defined as five sets of link elements with a diameter of 40 µm that connect the ciliary muscles and the lens to transfer the tension applied by the ciliary body (Fig. 1). As the precise anatomical location of the ligament remains to be conclusively identified, the proposed location was based on anatomical descriptions from literature.^10,15^ Therefore, the ligament of Wieger was modeled as a 20 µm thick ring-shaped membrane at three specific positions: the first and second had an inner diameter of 6 mm and a width of 0.5 mm or 1 mm respectively, while the third had an inner diameter of 7 mm and a width of 0.5 mm (Fig. 2). The anterior and vitreous chambers were not considered in the model to simplify the analysis and decrease the computational cost.

**Table 1:** Geometrical parameters for eye components in mm.

Central corneal radii of curvature ${R}_{c}$	7.80
Central corneal thickness	0.545
Anterior Limbal thickness	0.77
Limbal radius ${R}_{l}$	5.85
Posterior Limbal thickness	0.75
Scleral radius Rs	11.50
Scleral equatorial thickness	0.56
Anterior chamber depth	3.00
Iris thickness	0.30
Pupil diameter	2.60
Ciliary muscle length	4.60
Ciliary muscle thickness at apex	0.72
Ciliary muscle thickness at 25% length	0.54
Ciliary muscle thickness at 50% length	0.33
Ciliary muscle thickness at 75% length	0.16
Zonular fibers thickness	0.04
Lens thickness LT	4.20
Lens anterior radius of curvature	8.05
Lens posterior radius of curvature	5.05
Lens equatorial diameter	8.56
Cortex anterior thickness	0.80
Cortex posterior thickness	0.50
Lens equatorial radius	8.56
Lens nucleus anterior radius of curvature	4.00
Lens nucleus posterior radius of curvature	3.25
Lens capsule shell thickness	0.01
Ligament of Wieger thickness	0.02
Ligament of Wieger width	0.50 or 1.00
Ligament of Wieger distance from lens optical axis	3.00 or 3.50

### Material properties

Material properties were assigned to each structure based on literature values (Table 2). The first-order Ogden hyperelastic model was used to model the material behaviour of the cornea. The strain energy potential of this model is derived from the principal components of the left Cauchy-Green deformation tensor and is expressed as follows:

1 $$ W = \mathop \sum \limits_{i = 1} \frac{{\mu_{i} }}{{\alpha_{i} }}\left( {\overline{\lambda }_{1}^{{\alpha_{i} }} + \overline{\lambda }_{2}^{{\alpha_{i} }} + \overline{\lambda }_{3}^{{\alpha_{i} }} - 3} \right) + \mathop \sum \limits_{k = 1} \frac{1}{{D_{k} }}\left( {J - 1} \right)^{2k} $$

where *μ*_i_ and *α*_i_ are material constants and *D*_k_ is the incompressible parameter indicating volume changes. *J* is the volume ratio before and after deformation and for incompressible materials *J* = *1*.

**Table 2:** Material properties for eye components.

Cornea^16,17^	*μ* = 2.405 kPa, α = 101.878, 1/D = 10^7^ MPa, $\rho$ = 1225 kg/m^3^
Sclera-Limbus^16^	$C_{10}$ = 0.81 MPa, $C_{20}$ = 56.05 MPa, ${{\text{C}}}_{30}$ = 2332.26 MPa, 1/*D* = 10^7^ MPa, $\rho_{s}$ = 1225 kg/m^3^
Ciliary body^16^	*E* = 0.35 MPa, *ν* = 0.47, $\rho$ = 1225 kg/m^3^
Iris^18,19^	*E* = 3 kPa, *ν* = 0.47, $\rho$ = 1000 kg/m^3^
Zonules^16^	*E* = 0.35 MPa, *ν* = 0.47, $\rho$ = 1225 kg/m^3^
Lens nucleus^20,21^	*E* = 0.3 kPa, *ν* = 0.49
Lens cortex^20,21^	*E* = 3 kPa, *ν* = 0.49
Lens capsule^22^	*E* = 1000 kPa, *ν* = 0.49

The sclera and limbus were characterized using the Yeoh third order hyperelastic model, which accounts for the non-linear response of materials to different loading and strain conditions. The model is described as follows:

2 $$ W = \mathop \sum \limits_{i = 1}^{3} C_{i0} \left( {\tilde{I}_{1} - 3} \right)^{i} + \mathop \sum \limits_{i = 1}^{3} \frac{1}{{D_{{k_{i} }} }}\left( {J - 1} \right)^{2i} $$

Here, ${\widetilde{I}}_{1}$ represents the first invariant of the Cauchy-Green strain tensor, and ${C}_{{\text{i}}0}$ denotes the shear modulus that correlates with sclera behaviour.

Materials with elastic properties were chosen for the other components of the eye model. The response of these materials is commonly described by Hooke's law, which in its basic form is expressed as *σ* = *E*⋅*ϵ*, where *σ* is the stress, *E* is Young's modulus—a measure of the stiffness of the material—and *ϵ* is the strain, the deformation of the material relative to the applied stress.

### Mesh and boundary conditions

Considering the symmetry of the lens about its central axis and the negligible effect by the posterior segment of the eye on accommodation, it was deemed sufficient to simulate only an axisymmetric model of the anterior eye while significantly reducing computational costs (Fig. 1).

The model featured an axisymmetric triangular mesh, utilizing the PLANE183 element type for the cornea, sclera, limbus, iris, ciliary body, lens cortex, and nucleus, and all the connections between these components were bonded type. The SHELL208 element type was selected for the lens capsule, which was an axisymmetric shell as a surface coating for the cortex. Finally, the zonular fibers were meshed using the LINK180 element type with circular cross section. Ansys automatically generated this configuration, ensuring that each element type was compatible with the axisymmetric nature of the model.

**Figure 3:**
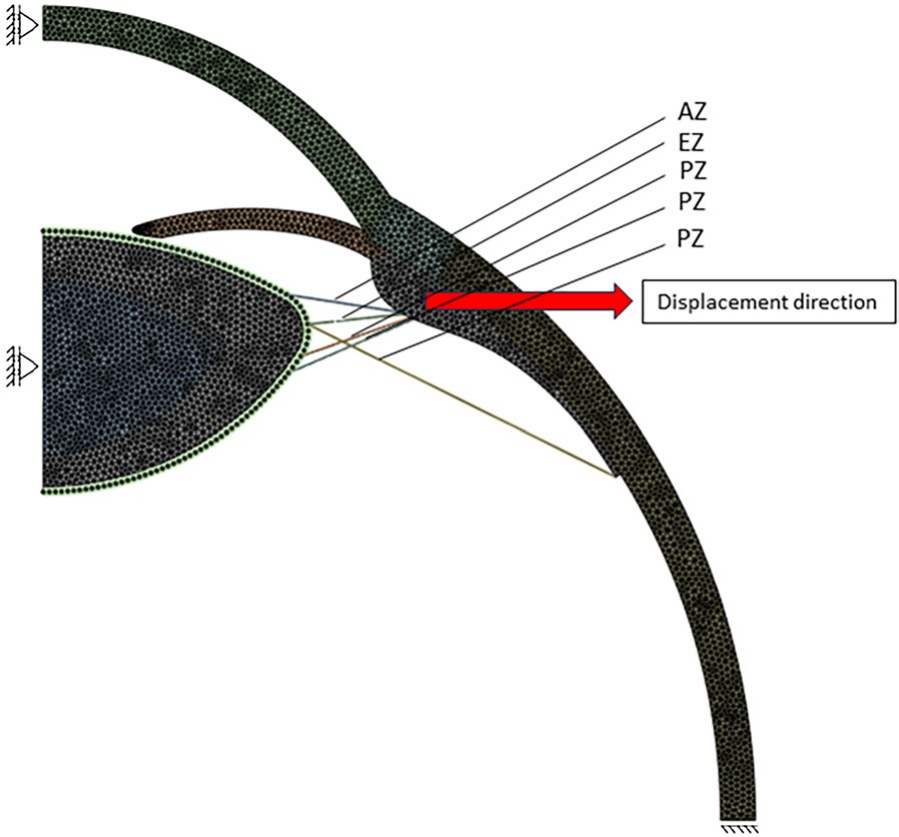
Indicating Model boundary conditions and zonular fibers, AZ (anterior zonula), EZ (equatorial zonula), PZ (posterior zonula).

To ensure appropriate modelling, we restricted the bottom of the sclera to prevent unwanted translations or rotations of the eye. We restricted movement of the cornea and lens to solely the optical axis. Regarding the Wieger ligament, we treated it as a component bonded to the lens capsule without adding any further boundary conditions (Fig. 3).

### Mesh convergence analysis

**Figure 4:**
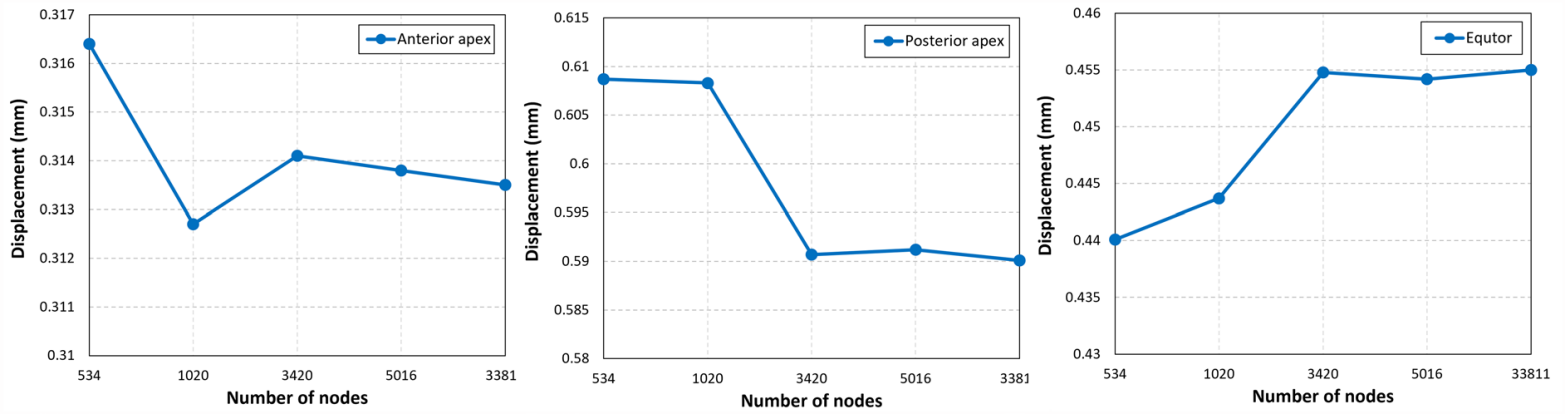
Graphical representation of displacement at the anterior apex, posterior apex, and equatorial region across different mesh densities. A mesh configuration with 3420 nodes and 7658 elements has been determined to be the minimum required to ensure convergence of the computational model, indicating the point at which further refinement does not result in significant changes to displacement outcomes.

A mesh sensitivity analysis was conducted to determine the optimal mesh size. Therefore, five models were generated, each with a different mesh density (Table 3). Our analysis revealed that the displacements at the equator, anterior pole, and posterior pole of the lens converged for the model containing 7658 elements and 3420 nodes (Fig. 4). Consequently, we selected this mesh density for the formal analysis in our study.

**Table 3:** Mesh analysis.

Mesh size (mm)	Number of nodes	Number of elements
0.3	534	1328
0.2	1020	2459
0.1	3420	7658
0.08	5016	11,066
0.03	33,811	70,389

### Simulation

Accommodation is controlled by the ciliary muscle contraction, which was simulated by displacing the muscle 0.5 mm away from the central axis (Fig. 3) in 8 steps, indicated as ‘levels of zonular stretching’ between 0 and 100%. This stretching was preferred over the accommodation as a reference since the presence of the ligament would affect the amount of accommodation that can be accomplished. Starting from the fully accommodated state with a power of 29.65D, zonular stretching gradually leads to the flat lenticular shape of the non-accommodated state. The effect of the ligament of Wieger on lens shape and power was investigated by repeating this process under several conditions. The first analysis was performed without the ligament to serve as a control, followed by four analyses with a ligament at different stiffness levels (half, once, twice, and three times the stiffness of the lens capsule) with a width of 0.5 mm and an inner diameter of 6 mm (marked as ‘0.5W6D’). Two additional analyses were conducted to evaluate the impact of ligament width and inner diameter. The first involved a ligament with a width of 1 mm and an inner diameter of 6 mm (‘1W6D’), while the second used ligaments with a width of 0.5 mm and an inner diameter of 7 mm (‘0.5W7D’).

The nodal coordinates after deformation were extracted from the anterior and posterior lens surface within 5 mm diameter in optical zone from the model for each analysis and used to determine the lens surface radii of curvature (${r}_{{\text{a}}}$ and ${r}_{{\text{p}}}$) by post processing analysis in MATLAB (R2023a, The Mathworks, Natick, MA, USA) and Excel (v365, Microsoft, Seattle, WA, USA). To do this, both lens surfaces were fitted by an aspherical surface given by:

3 $$Z\left(x\right)= a{x}^{2}\left(1+\sqrt{1-(1+b){a}^{2}{x}^{2}}\right)$$

where $x$ is the radial distance from the optical axis and a is curvature and b is conic constant. These values were used to calculate the anterior, posterior, and total power with the following formulas:

4 $${P}_{{\text{anterior}}}=\frac{{n}_{{\text{l}}}-{n}_{{\text{a}}}}{{r}_{{\text{a}}}}1000$$

5 $${P}_{{\text{posterior}}}=\frac{{n}_{{\text{a}}}-{n}_{{\text{l}}}}{{r}_{{\text{a}}}}1000$$

6 $$ P_{{{\text{total}}}} = P_{{{\text{anterior}}}} + P_{{{\text{posterior}}}} - P_{{{\text{anterior}}}} \cdot P_{{{\text{posterior}}}} \cdot \frac{LT}{{1000 \cdot n_{{\text{l}}} }} $$

with *LT*, the lens thickness (in meter), ${r}_{{\text{a}}}$ and ${r}_{{\text{p}}}$ the lens radii of curvature (in meter), *n*_l_ = 1.43, the equivalent lens refractive index, and *n*_a_ = 1.336, the refractive index of the aqueous humor. In practice, the refractive index of 1.42 given by Burd et al.^14^ was found to give lens power values that were unrealistically low. Hence, the refractive index from the SyntEyes model^23^ was used instead.

The total force exerted by the zonular fibers was determined using ANSYS by extracting the forces of each zonular fiber directly and then adding these values to obtain the overall zonular force.

## Results

On a macroscopic scale, the influence of the ligament of Wieger on the non-accommodated lens shape appears modest, but the optical consequences would be noticeable.

### Influence of ligament stiffness

**Figure 5:**
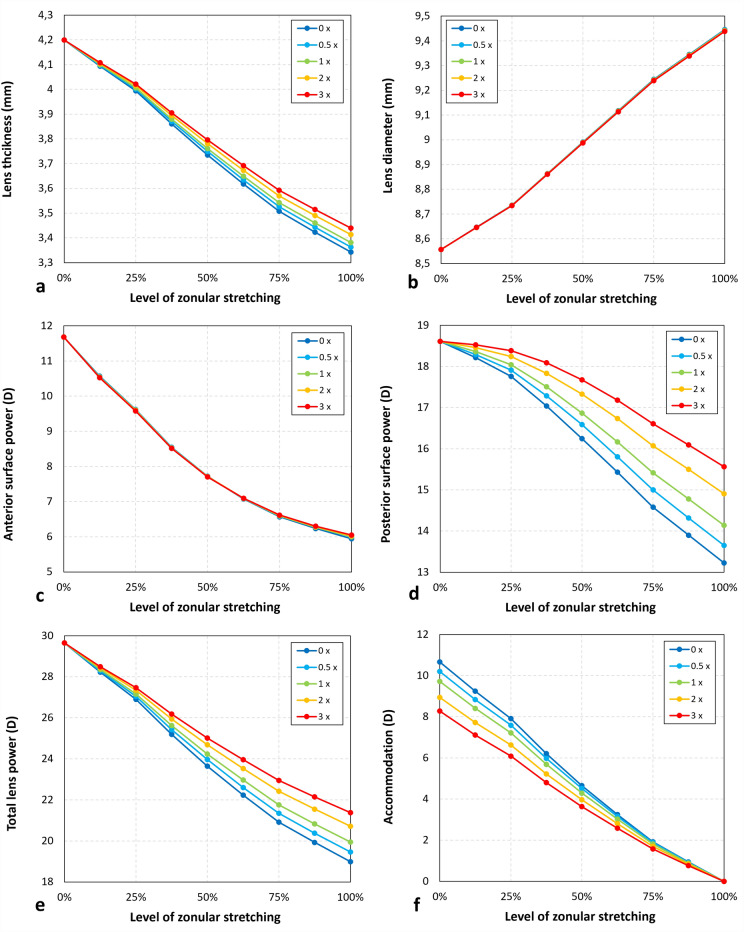
Changes in parameters describing the shape of the crystalline lens for various accommodative states, ranging between full accommodation (0% zonular stretching) and no accommodation (100% zonular stretching), and various values of ligament stiffness (in multiples of capsular bag stiffness). (**a**) Lens thickness; (**b**) Lens diameter; (**c**) Anterior surface power; (**d**) Posterior surface power; (**e**) Total lens power; (**f**) Accommodation.

For a ligament with the same stiffness as the lens capsule, the non-accommodated lens thickness increased by 38 μm and by 97 μm for the highest stiffness (Fig. 5a). Lens diameter and the anterior surface power saw only minor changes for the highest level of ligament stiffness with increases of 0.007 mm and 0.1D, respectively, (Fig. 5b,c). As expected, the non-accommodated posterior surface power is most affected by the ligament, with increases of 0.92D and 2.34D for ligaments with the same and three times the stiffness of the capsular bag (Fig. 5d). Very similar values are found for the changes in total lens power (Fig. 5e). Since the accommodative range can be defined as the difference in lens power between the fully accommodated and non-accommodated states, an increase in the latter while the former remains constant will inevitably affect the range by − 0.95D and − 2.39D for ligaments with the same and three times the stiffness of the capsular bag (Fig. 5f).

### Influence of ligament width and inner diameter

**Figure 6:**
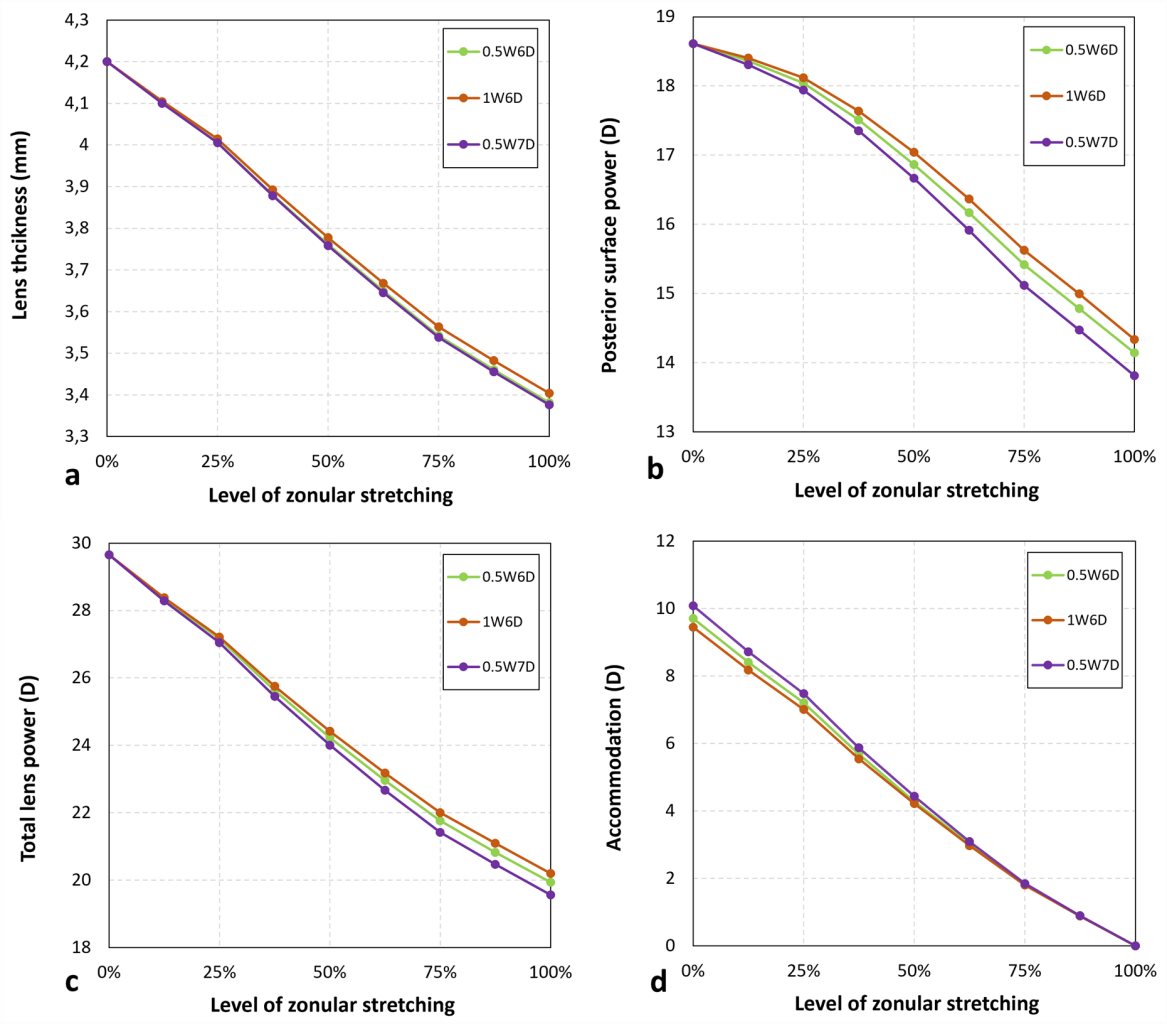
Changes in parameters describing the shape of the crystalline lens.

To assess the influence of the width and inner diameter model 0.5W6D with the same stiffness as the capsular bag was considered as a reference. Increasing the inner diameter to 7 mm (0.5W7D) had only a negligible effect on the non-accommodated less thickness states, while a broader ligament led to a minor increase in thickness by 22 µm (Fig. 6a). There was, however, a noticeable difference in posterior surface power, with changes of + 0.19D for 1W6D and − 0.33D for 0.5W7D (Fig. 6b). The corresponding changes in accommodation were − 0.26D and + 0.37D, respectively (Fig. 6c,d).

### Zonular forces

**Figure 7:**
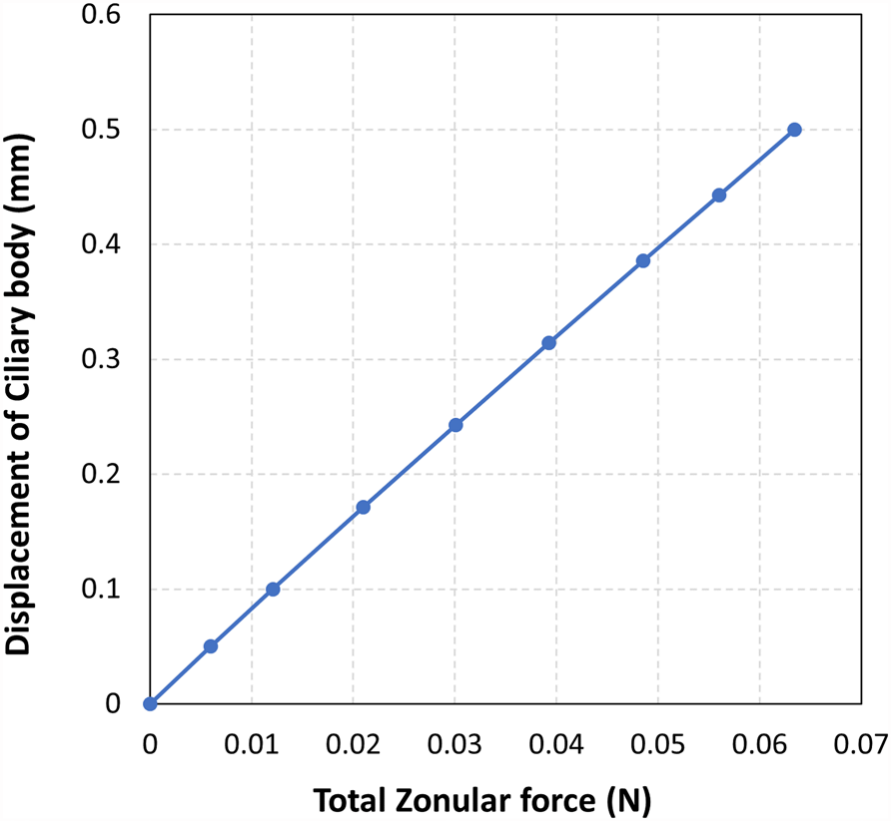
Zonular forces in the model without the ligament of Wieger.

At maximum accommodation the total force exerted by the zonular fibers was 0.064 N (Fig. 7). This is in close agreement with the findings of a study using a 29-year-old lens model, where the average total net force from the zonular fibers and ciliary muscle was reported as 0.056 N^24^. Additionally, our result aligns with the force of 0.055 N recorded in an in vitro stretching experiment on a 47-year-old human eye^25^, further corroborating the reliability and relevance of our simulation data.

## Discussion

Not much is known about the ligament of Wieger is a difficult structure to observe and measure in vivo. Under certain conditions, however, such as in pigment dispersion syndrome, where Scheie's line may appear^26–28^, trauma^29^, or intraocular hemorrhage^30^ the normally transparent structure may become visible. The exact function of the ligament is not fully understood. Initially, it was believed to be a vestigial remnant of the fetal hyaloid vasculature, but it has since been suggested that it may play a role in accommodation^11^. Building on this, Goldberg theorized that the ligament of Wieger cradles the lens to hold it in place using additional zonules, although the existence of these zonules is yet to be confirmed.

Our simulations show that the presence of the ligament, attached to the posterior lens surface, affects both the shape of the non-accommodated lens and the accommodative range in the model (Fig. 5). These effects become more important for higher levels of ligament stiffness, although the actual stiffness remains unknown. The width and inner diameter of the ligament of Wieger also have a smaller, but noticeable impact on the optical properties of the lens. These findings are consistent with those observed by Ljubimova et al.^11^, who suggested that the inclusion of the ligament leads to smaller changes in the posterior radius of curvature, indicating increased rigidity in the posterior lens surface. Moreover, our results demonstrate that changes in central thickness and curvatures with accommodation also reduced in the presence of the ligament compared to when it was not included.

Another important aspect to consider is the strength of the adhesion between the ligament and the capsular bag. This is thought to be strong in young eyes, but gradually weakens with age until the ligament detaches, as is seen clinically in many individuals over the age of 65 years^31^. In this model, such a detachment would appear as a decrease in posterior capsular stiffness. Given that the posterior lens curvature does not change (much) during adulthood^32^, as opposed to the anterior curvature that sees large changes, one may speculate that the presence of the ligament preserves the posterior lens shape. But since the crystalline lens fibers never stop dividing^33^, thus increasing the thickness and curvature of the lens, it is conceivable that stress gradually increases at the area where the ligament is attached to the capsule, leading to their eventual separation. This gradual detachment reduces the ligament’s influence on the posterior lens shape, allowing it to deform more during accommodation, which may temporarily dampen the effects of presbyopia. This is speculative, however, until further in vivo data can be obtained. These findings highlight the importance of further investigating the role of the ligament of Wieger in age-related changes in the lens and may have implications for the development of future treatments for presbyopia.

As with any model, it is important to note that it is based on several assumptions to approximate the structure of the eye, which may have had some minor consequences for the model’s accuracy. One such assumption was that the ligament was attached in the fully relaxed state. Alternatively, one could attach it in the fully stretched state, but then the lens would not be able to reach the same level of full accommodation. When attached in an in-between state, it would affect both the relaxed and accommodated states, influencing the range of accommodation even further. Furthermore, the model assumes that the accommodation works in reverse, from a relaxed to a non-relaxed state, but this should not affect the results. Age could be a factor as well since some studies^34,35^ indicate that in young lenses the shear modulus of the nucleus could be as high as or higher than that of the cortex, which could affect the model's predictions. Other possible factors include the variations in capsular thickness into the model, which could have slightly alter the results, or conditions affecting the adhesion of the Wieger ligament, such as diabetes^36^. Note also that the mechanical influences of the ligament presented in this work greatly depend on its unknown stiffness. This was addressed by considering a broad range of stiffness values that likely includes that correct value, as well as values that are too high or too low. Finally, the model does not consider the effects of fluidic transmitter forces that would have slightly dampened accommodation.

In conclusion, the results of this analysis suggest that ligament of Wieger has a noticeable effect on lens shape and accommodation that is important enough to merit consideration when biomechanically modelling these processes.

## Data Availability

Data is available from Prof. Jos Rozema upon reasonable request.
